# Direct Comparison of Three Postpartum Hemorrhage Risk Assessment Tools: A Retrospective Secondary Data Analysis

**DOI:** 10.1155/ogi/9576600

**Published:** 2026-05-21

**Authors:** Stefanie Modri, Mohika Nagpal, Margaret Brace, Yvonne Nicole Reddick, James Weimer, Harish Sehdev, Kimberly Kovach Trout

**Affiliations:** ^1^ Department of Family & Community Health, University of Pennsylvania, Philadelphia, Pennsylvania, USA, upenn.edu; ^2^ University of Michigan, Ann Arbor, Michigan, USA, umich.edu; ^3^ M. Louise Fitzpatrick College of Nursing, Villanova, Pennsylvania, USA; ^4^ Hospital of the University of Pennsylvania, Philadelphia, Pennsylvania, USA, pennmedicine.org; ^5^ Institute for Surgery and Engineering, Vanderbilt University, Nashville, Tennessee, USA, vanderbilt.edu; ^6^ Perelman School of Medicine and Pennsylvania Hospital, University of Pennsylvania, Philadelphia, Pennsylvania, USA, upenn.edu; ^7^ M. Louise Fitzpatrick College of Nursing and Pennsylvania Hospital, Philadelphia, Pennsylvania, USA

**Keywords:** anemia, childbirth, maternal morbidity, midwifery, obstetrics, postpartum hemorrhage

## Abstract

**Objective:**

The objective was to compare the performance of three postpartum hemorrhage risk screening tools with a known patient data set.

**Design:**

This is a retrospective secondary data analysis of a parent study whose aim was to develop a novel biomarker for detecting elevated blood loss with childbirth.

**Setting:**

A single tertiary care hospital in the United States.

**Participants:**

Inclusion criteria for the parent study were ≥ 37 weeks, ≥ 18 years, and pregnancy with one live fetus (no multiples). Eligible participants were identified upon admission to the labor unit and enrolled after informed consent was obtained.

**Methods/Main Outcome Measures:**

Statistical analyses were computed using Stata SE, v18. Bivariate associations between binary outcomes and continuous measures were calculated using independent *t*‐tests. Bivariate associations between binary outcomes and categorical measures were calculated using chi‐square tests.

**Results:**

Recruitment occurred June through September 2021 and included *n* = 525 participants. When using the American College of Obstetricians and Gynecologists’ threshold of ≥ 1000 mL, 13.3% (n = 70) of parturients experienced a hemorrhage. When using the World Health Organization’s definition of postpartum hemorrhage of ≥ 500 mL, the prevalence of hemorrhage was 36.8% (*n* = 193). Sensitivity was highest in identifying hemorrhage with the Association of Women’s Health, Obstetric and Neonatal Nurses’ tool. When high‐ and medium‐risk scores were considered, the tool had 86% (95% CI: 83.0%–89.0%) accuracy in identifying cases of hemorrhage. This tool also had the highest negative predictive value (78.7%, CI 75.2%–82.2%).

**Conclusions:**

The tool with the highest sensitivity and negative predictive values identified 21% of cases as “low risk” that went on to experience blood loss ≥ 500 mL, thus underscoring the need for better predictive models.

## 1. Introduction

Postpartum hemorrhage (PPH) is an obstetrical emergency that persists as the leading cause of global maternal mortality [1]. While more deaths occur in low‐ and middle‐income countries (LMICs), PPH contributes significantly to maternal deaths and severe maternal morbidity in high‐income countries as well [2]. To address this crisis, the World Health Organization (WHO) has produced a WHO Roadmap to Combat Postpartum Hemorrhage 2023 to 2030 that underscores the importance of facility preparedness, norms of care, and implementation guidelines [3]. Prediction and preparedness are important factors to reduce the severity of PPH [4, 5]. In the United States, there are three main PPH risk assessment screening tools used, developed by the California Maternal Quality Care Collaborative (CMQCC) [6], the American College of Obstetricians and Gynecologists (ACOG) [7, 8], and the Association of Women’s Health, Obstetric and Neonatal Nurses (AWHONN) [9], respectively. These tools are designed to stratify patients into low‐, medium‐, and high‐risk categories for PPH, thereby preparing birthing teams to curtail blood loss via timely delivery of uterotonics and other therapies. Procedures for documenting this risk will differ among various institutions, but PPH risk scoring upon admission to the hospital for childbirth is a Joint Commission standard and is typically documented in the medical record. Consistent shortcomings of these risk assessment tools are their low to moderate sensitivity and specificity [10–13]. Assessing these tools in separate studies, with different patient populations, and with varying outcome measures makes direct comparison of the tools difficult.

The primary aim of this study was to determine the comparative accuracies, predictive powers, sensitivities, and specificities of the CMQCC, the ACOG, and the AWHONN tools when compared with a known patient data set.

## 2. Methods

The current study is a retrospective secondary data analysis. The parent study was a prospective, observational study that used convenience sampling of eligible participants who were recruited from June through September 2021. This study was conducted at a tertiary care hospital in the city of Philadelphia (United States). Prior to the performance of any study procedures, ethics approval was obtained from the University of Pennsylvania Institutional Review Board # 848588 on May 25, 2021. The aim of the parent study was to identify a biomarker and develop an algorithm based on maternal heart rate data that would indicate parturients at increased risk for excessive blood loss [14].

### 2.1. Participants

Eligibility included the following: ≥ 37 weeks gestation, 18 years or older, pregnancy with one live fetus (no multiples, no intrauterine fetal demise). Potential participants were identified upon admission to the obstetrical unit through review of the electronic medical record to determine if they met inclusion criteria for the primary study. Written, informed consent was obtained prior to any study participation. Enrollment included parturients of diverse ethnic and racial backgrounds to ensure that the benefits and burdens of research were equitably distributed.

### 2.2. Procedures

Chart abstracts were completed for data points related to the specific aims. Individual criteria were placed into the AWHONN, CMQCC, and ACOG PPH risk assessment tools to determine their level of PPH risk (low, medium, or high) by an independent rater not involved in the clinical care of the patient. While this institution uses an adapted version of the AWHONN tool, the independent raters did not have knowledge of, and therefore, did not use the AWHONN risk determination used for clinical care. Hemoglobin levels were dichotomized into medium/high (< 10) versus low (≥ 10) risk, as defined by the ACOG risk tool. Total blood loss was documented in the medical record of each participant as either an estimated blood loss (EBL, 96% of cases) or a quantitative blood loss (QBL, 4% of cases) and was recorded by the birth attendant. Study data were collected and managed using the Research Electronic Data Capture (REDCap) system, a secure, web‐based application for collecting and managing research data [15]. All health information was entered into REDCap by research assistants and then double‐checked for accuracy by registered nurses.

### 2.3. Statistical Analysis

Analyses were computed for all participants entering the study using Stata SE, version 18 (Stata Corp: College Station, TX). The data were examined to determine the presence of marked skewness, outliers, and systematic missing data. Data were complete for all risk assessment tools and for medication administration. One case was missing data about blood loss volume and was excluded from analyses pertaining to blood loss. Across all tests, an alpha of < 05 and 95% confidence intervals were used to assess statistical significance.

Demographic and clinical characteristics of the sample were summarized using means and standard deviations for continuous measures, or frequencies and percentages for categorical variables. Bivariate associations between binary outcomes and continuous measures were calculated using independent *t*‐tests. Bivariate associations between binary outcomes and categorical measures were calculated using chi‐square tests or, if expected cell counts were less than five, Fisher’s exact tests.

The sensitivity, specificity, positive predictive value, and negative predictive value were calculated for each combination of risk assessment tool and outcome using standard methods for assessing diagnostic tools in 2 × 2 tables (tool assessment × observed outcome). Since the CMQCC, ACOG, and AWHONN rate patients as low, medium, or high risk, two interpretive possibilities were considered. Initially, we assessed the sensitivity, specificity, and predictive values of just the “high risk” designation for each tool. We then considered the sensitivity, specificity, and predictive values for “medium risk” and “high risk” combined.

The similarity of risk assessment scales was assessed using Spearman’s rank correlation coefficient and percent agreement. To evaluate the predictive utility of the risk assessment screening tools, dichotomous clinical outcomes (uterotonic medication, blood loss ≥ 500 mL, blood loss ≥ 1000 mL, and hemoglobin < 10.0) were regressed on each risk assessment tool score using generalized linear models with log links to produce crude, unadjusted estimates of the association between screening tool assessments and clinical outcomes. No additional covariates were included in these models, since the purpose of the analysis was to study the predictive utility of each of the screening tools and not to create new predictive algorithms that include supplemental information. Receiver operating characteristic (ROC) curves were then graphed, and areas under the curve were computed, for each risk assessment tool and each outcome. Calibration plots were also generated to show the proportion of participants experiencing each of the clinical outcomes across the tool‐specified risk levels.

Finally, because the AWHONN tool was used by clinicians providing care, this incorporation bias might inflate the performance of this tool for the administration of non‐prophylactic medication. Therefore, analyses were replicated on the sample of participants who did not receive medication (*n* = 386) as a sensitivity analysis.

## 3. Results

A total of *n* = 525 patients were enrolled in the study. Blood loss data were missing for one participant; therefore, for analyses pertaining specifically to blood loss, *n* = 524. When PPH is defined as blood loss ≥ 500 mL per WHO [16], 36.8% of the sample (*n* = 193) experienced PPH. When PPH is defined as blood loss ≥ 1000 mL per ACOG [7], 13.3% of the sample (*n* = 70) experienced PPH.

The mean age of participants was 31.7 years (SD 4.9) with a mean gestation of 39.2 weeks (SD 1.1). Other demographic and clinical characteristics of the sample are summarized in Table 1. Twenty‐six percent of participants (*n* = 139) received at least one uterotonic medication beyond prophylactic oxytocin (Pitocin). Participants who received medications were similar to those who did not when assessed for age, gestational age, race, ethnicity, or insurance type. Those who received medications had a higher average BMI (*p* = 0.045), a higher proportion of cesarean births (*p* = 0.014) and were more likely to be primiparous (*p* < 0.001).

**TABLE 1:** Demographic and clinical characteristics of the study participants.

	**Total**		**Outcome: PPH medication**					**Outcome: blood loss**				
	**Sample**		**None**		**One +**		**p** **∗**	**< 1000 mL**		**≥ 1000 mL**		**p** **∗**
	***n* = 525**		***n* = 386, 74%**		***n* = 139, 26%**			***n* = 442, 84%**		***n* = 82, 16%**		
Mother’s age, years	31.7	(4.9)	31.7	(5.0)	31.8	(4.6)	0.948	31.7	(4.9)	32.1	(4.5)	0.506
Gest. age, weeks	39.2	(1.1)	39.1	(1.1)	39.2	(1.1)	0.408	39.2	(1.1)	39.3	(1.1)	0.417
BMI	32.4	(6.4)	32.1	(6.5)	33.4	(6.1)	**0.045**	32.1	(6.1)	34.3	(7.5)	**0.005**
Race												
Black	132	25.1%	96	24.9%	36	25.9%	0.628	108	24.4%	24	29.3%	0.404
Unknown	65	12.4%	51	13.2%	14	10.1%		58	13.1%	7	8.5%	
White	328	62.5%	239	61.9%	89	64.0%		276	62.4%	51	62.2%	
Ethnicity												
Hispanic or Latino	54	10.5%	37	9.8%	17	12.4%	0.368	43	9.9%	11	13.4%	0.345
Not Hispanic	462	89.5%	342	90.2%	120	87.6%		390	90.1%	71	86.6%	
Type of insurance												
Public	141	27.1%	99	25.9%	42	30.4%	0.306	113	25.9%	28	34.1%	0.122
Private	379	72.9%	283	74.1%	96	69.6%		324	74.1%	54	65.9%	
Delivery type												
Vaginal	359	68.4%	276	71.5%	83	59.7%	**0.014**	340	76.9%	18	22.0%	**< 0.001**
Cesarian	142	27.0%	90	23.3%	52	37.4%		78	17.6%	64	78.0%	
VAVD	12	2.3%	10	2.6%	2	1.4%		12	2.7%	0	0.0%	
TOLAC or VBAC	12	2.3%	10	2.6%	2	1.4%		12	2.7%	0	0.0%	
Parity												
0	265	50.5%	180	46.6%	85	61.2%	**< 0.001**	218	49.3%	47	57.3%	**0.035**
1	153	29.1%	126	32.6%	27	19.4%		138	31.2%	14	17.1%	
2	68	13.0%	57	14.8%	11	7.9%		57	12.9%	11	13.4%	
3 or more	39	7.4%	23	6.0%	16	11.5%		29	6.6%	10	12.2%	
WHO: PPH												
< 500 mL blood loss	331	63.2%	283	73.5%	48	34.5%	**< 0.001**	331	74.9%	0	0.0%	**< 0.001**
≥ 500 mL loss	193	36.8%	102	26.5%	91	65.5%		111	25.1%	82	100.0%	
ACOG: PPH												
< 1000 mL blood loss	442	84.4%	358	93.0%	84	60.4%	**< 0.001**	—	—	—	—	
≥ 1000 mL loss	82	15.6%	27	7.0%	55	39.6%		—	—	—	—	
PPH medication												
None	386	73.5%	—	—	—	—		358	81.0%	27	32.9%	**< 0.001**
Any	139	26.5%	—	—	—	—		84	19.0%	55	67.1%	
CMQCC												
Low risk	325	61.9%	261	67.6%	64	46.0%	**< 0.001**	295	66.7%	30	36.6%	**< 0.001**
Medium risk	128	24.4%	85	22.0%	43	30.9%		99	22.4%	28	34.2%	
High risk	72	13.7%	40	10.4%	32	23.0%		48	10.9%	24	29.3%	
ACOG												
Low risk	350	66.7%	272	70.5%	78	56.1%	**< 0.001**	311	70.4%	39	47.6%	**< 0.001**
Medium risk	101	19.2%	73	18.9%	28	20.1%		78	17.7%	22	26.8%	
High risk	74	14.1%	41	10.6%	33	23.7%		53	12.0%	21	25.6%	
AWHONN												
Low risk	127	24.2%	109	28.2%	18	13.0%	**< 0.001**	121	27.4%	6	7.3%	**< 0.001**
Medium risk	235	44.8%	175	45.3%	60	43.2%		196	44.3%	39	47.6%	
High risk	163	31.1%	102	26.4%	61	43.9%		125	28.3%	37	45.1%	
Hemoglobin risk												
Low: Hgb ≥ 10	496	94.5	366	94.8%	130	93.5%	0.567	425	96.2%	70	85.4%	**< 0.001**
High: Hgb < 10	29	5.5%	20	5.2%	9	6.5%		17	3.9%	12	14.6%	

*Note:* Means and standard deviations, or frequencies and percentages, are presented for the total sample (*n* = 525), and are compared across two outcomes: (1) whether study participants were or were not administered uterotonics beyond prophylactic oxytocin (*n* = 525), and (2) whether study participants experienced blood loss < 1000 mL or ≥ 1000 mL (*n* = 524). Compar. = Comparison; Hgb = hemoglobin. Bold values represent statistical significance.

Abbreviations: ACOG = American College of Obstetricians and Gynecologists, PPH = post‐partum hemorrhage, TOLAC = trial of labor after cesarean, VAVD = vacuum‐assisted vaginal delivery; VBAC = vaginal birth after cesarean; WHO = World Health Organization.

^∗^Comparisons based on independent sample *t*‐tests for continuously measured variables, chi squared tests for categorical variables, or Fisher’s exact test when expected cell counts are less than 5 (Delivery Type). Cells do not sum to 525 in some cases due to missing data.

Similar to the patterns observed with uterotonic use, those experiencing ≥ 1000 mL blood loss had significantly higher mean BMI (*p* = 0.005), more commonly had a cesarean birth (*p* < 0.001), and were more commonly primiparous (*p* = 0.035) than those experiencing lower levels of blood loss.

In this sample, the AWHONN tool classified more cases as medium or high risk, when compared with CMQCC and ACOG (Table 1). CMQCC and ACOG ratings are most closely associated (Spearman rank correlation coefficient = 0.776, *p* < 0.001); they agree in their ratings for 82.3% of cases (Table 2). While most of the disagreement between the scales occurs when cases rated as low or high risk by one scale are rated as medium risk by the other, there are 1% of cases (*n* = 6) where the disagreement is more extreme, with the patient rated as high risk by one of these scales and low risk by the other. The AWHONN risk assessment tool is somewhat less correlated with the CMQCC (Spearman rank correlation 0.563, *p* < 0.001; 47.6% agreement) and the ACOG (Spearman rank correlation 0.571, *p* < 0.001; 46.1% agreement).

**TABLE 2:** Correlations and percent agreement for the CMQCC, ACOG, and AWHONN risk assessments, as well as hemoglobin level (*n* = 525).

	CMQCC	ACOG	AWHONN	Hemoglobin risk
CMQCC				
Spearman corr.	1	—	—	—
*p* value	—	—	—	—
% Agreement	100%	—	—	—
ACOG				
Spearman corr.	0.776	1	—	—
*p* value	< 0.001	—	—	—
% Agreement	82.3%	100%	—	—
AWHONN				
Spearman corr.	0.563	0.571	1	—
*p* value	< 0.001	< 0.001	—	—
% Agreement	47.6%	46.1%	100%	—
Hemoglobin risk^∗^				
Spearman corr.	0.300	0.327	0.090	1
*p* value	< 0.001	< 0.001	0.039	—
% Agreement	86.5%	86.5%	68.4%	100%

*Note:* Spearman Corr. = Spearman rank correlation coefficient.

Abbreviations: ACOG = American College of Obstetricians and Gynecologists, AWHONN = Association of Women’s Health, Obstetrics and Neonatal Nurses, CMQCC = California Maternal Quality Care Collaborative.

^∗^Correlation and agreement for the dichotomous hemoglobin risk and other risk scales is calculated using dichotomized versions of the CMQCC, ACOG, and AWHONN (low or medium risk, vs. high risk).

The hemoglobin risk indicator was dichotomous, with hemoglobin levels < 10 indicating higher risk for PPH. Table 2 shows the association and agreement between hemoglobin levels and the other three risk assessment tools when they are dichotomized to “low or medium” and “high” risk groups. The dichotomized CMQCC and ACOG are similar in their level of agreement with the hemoglobin risk assessment (86.5% each). The dichotomized AWHONN and hemoglobin levels are concordant only 68.4% of the time.

Important outcome measures were administration of at least one uterotonic medication (beyond prophylaxis for active management of third stage of labor) or documentation of blood loss at the two volume thresholds. To understand how robust each of the three risk assessment tools’ predictive powers are, we compared the risk assessment tools with these indicators. Tables 3, 4, and 5 present the sensitivity, specificity, and predictive values of the three risk assessment tools hemoglobin, along with 95% confidence intervals for those calculations, across the three observed clinical outcomes (hemoglobin risk indicator results are presented in Supporting Table S1).

**TABLE 3:** Sensitivity, specificity, and predictive values of the CMQCC screening tool, for predicting uterotonic medication administration (*n* = 525), and blood loss volume (*n* = 524).

**CQMCC**	**Uterotonic medication**		**Blood loss ≥ 500 mL**		**Blood loss ≥ 1000 mL**	
	**%**	**(95% CI)**	**%**	**(95% CI)**	**%**	**(95% CI)**
*High risk*						
Sensitivity	23.0	(19.4–26.6)	22.3	(18.7–25.8)	29.3	(25.4–33.2)
Specificity	89.6	(87.0–92.2)	91.2	(88.8–93.7)	89.1	(86.5–91.8)
PPV	44.4	(40.2–48.7)	59.7	(55.5–63.9)	33.3	(29.3–37.4)
NPV	76.4	(72.8–80.0)	66.8	(62.8–70.9)	87.2	(84.3–90.0)

*High or medium risk*						
Sensitivity	54.0	(49.7–58.2)	59.1	(54.9–63.3)	63.4	(59.3–67.5)
Specificity	67.6	(63.6–71.6)	74.3	(70.6–78.1)	66.7	(62.7–70.8)
PPV	37.5	(33.4–41.6)	57.3	(53.1–61.5)	26.1	(22.4–29.9)
NPV	80.3	(76.9–83.7)	75.7	(72.0–79.4)	90.8	(88.3–93.3)

	**RR**	**(95% CI)**	**RR**	**(95% CI)**	**RR**	**(95% CI)**

*GLM*						
(ref: Low)						
Medium risk	1.7	(1.2–2.4)	2.3	(1.8–2.9)	2.4	(1.5–3.8)
High risk	2.3	(1.6–3.2)	2.5	(1.9–3.2)	3.6	(2.3–5.8)

*Note:* Sensitivity = percent of patients with the outcome who were designated “at risk” by the tool. Specificity = percent of patients without the outcome who were designated “not at risk” by the tool. Positive predictive value = percent of patients designated “at risk” by the tool who experienced the outcome. Negative predictive value = percent of patients designated “not at risk” by the tool who did not experience the outcome. Generalized linear models with log link for the regression of each observed clinical outcome on the risk assessment score are also presented. The table contains estimates and 95% confidence intervals. RR = relative risk (aka risk ratio); ref. = reference.

Abbreviations: 95% CI = 95% confidence interval, CMQCC = California Maternal Quality Care Collaborative, GLM = Generalized Linear Model, NPV = negative predictive value, PPV = positive predictive value.

**TABLE 4:** Sensitivity, specificity, and predictive values of the ACOG screening tool, for predicting uterotonic medication administration (*n* = 525), and blood loss volume (*n* = 524).

**ACOG**	**Uterotonic medication**		**Blood loss ≥ 500 mL**		**Blood loss ≥ 1000 mL**	
	**%**	**(95% CI)**	**%**	**(95% CI)**	**%**	**(95% CI)**
*High risk*						
Sensitivity	23.7	(20.1–27.4)	23.8	(20.2–27.5)	25.6	(21.9–29.4)
Specificity	89.4	(86.7–92.0)	91.5	(89.2–93.9)	88.0	(85.2–90.8)
PPV	44.6	(40.3–48.9)	62.2	(58.0–66.3)	28.4	(24.5–32.2)
NPV	76.5	(72.9–80.1)	67.3	(63.3–71.4)	86.4	(83.5–89.4)

*High or medium risk*						
Sensitivity	43.9	(39.6–48.1)	50.8	(46.5–55.1)	52.4	(48.2–56.7)
Specificity	70.5	(66.6–74.4)	77.0	(73.4–80.6)	70.4	(66.5–74.3)
PPV	34.9	(30.8–38.9)	56.3	(52.1–60.6)	24.7	(21.0–28.4)
NPV	77.7	(74.2–81.3)	72.9	(69.1–76.7)	88.9	(86.2–91.6)

	**RR**	**(95% CI)**	**RR**	**(95% CI)**	**RR**	**(95% CI)**

*GLM*						
(ref: Low)						
Medium risk	1.2	(0.9–1.8)	1.9	(1.5–2.5)	2.0	(1.2–3.2)
High risk	2.0	(1.5–2.8)	2.3	(1.8–2.9)	2.5	(1.6–4.1)

*Note:* Sensitivity = percent of patients with the outcome who were designated “at risk” by the tool. Specificity = percent of patients without the outcome who were designated “not at risk” by the tool. Positive predictive value = percent of patients designated “at risk” by the tool who experienced the outcome. Negative predictive value = percent of patients designated “not at risk” by the tool who did not experience the outcome. Generalized linear models with log link for the regression of each observed clinical outcome on the risk assessment score are also presented. The table contains estimates and 95% confidence intervals. RR = relative risk (aka risk ratio), ref. = reference.

Abbreviations: 95% CI = 95% confidence interval, ACOG = American College of Obstetricians and Gynecologists, GLM = Generalized Linear Model, NPV = negative predictive value, PPV = positive predictive value.

**TABLE 5:** Sensitivity, specificity, and predictive values of the AWHONN screening tool, for predicting uterotonic medication administration (*n* = 525), and blood loss volume (*n* = 524).

**AWHONN**	**Uterotonic medication**		**Blood loss ≥ 500 mL**		**Blood loss ≥ 1000 mL**	
	**%**	**(95% CI)**	**%**	**(95% CI)**	**%**	**(95% CI)**
*High risk*						
Sensitivity	43.9	(39.6–48.1)	42.0	(37.7–46.2)	45.1	(40.9–49.4)
Specificity	73.6	(69.8–77.4)	75.5	(71.9–79.2)	71.7	(67.9–75.6)
PPV	37.4	(33.3–41.6)	50.0	(45.7–54.3)	22.8	(19.3–26.4)
NPV	78.5	(74.9–82.0)	69.1	(65.1–73.0)	87.6	(84.7–90.4)

*High or medium risk*						
Sensitivity	87.1	(84.2–89.9)	86.0	(83.0–89.0)	92.7	(90.5–94.9)
Specificity	28.2	(24.4–32.1)	30.2	(26.3–34.1)	27.4	(23.6–31.2)
PPV	30.4	(26.5–34.3)	41.8	(37.6–46.0)	19.1	(15.8–22.5)
NPV	85.8	(82.8–88.8)	78.7	(75.2–82.2)	95.3	(93.5–97.1)

	**RR**	**(95% CI)**	**RR**	**(95% CI)**	**RR**	**(95% CI)**

*GLM*						
(ref: Low)						
Medium risk	1.8	(1.1–2.9)	1.7	(1.2–2.5)	3.5	(1.5–8.1)
High risk	2.6	(1.6–4.2)	2.4	(1.6–3.4)	4.8	(2.1–11.1)

*Note:* Sensitivity = percent of patients with the outcome who were designated “at risk” by the tool. Specificity = percent of patients without the outcome who were designated “not at risk” by the tool. Positive predictive value = percent of patients designated “at risk” by the tool who experienced the outcome. Negative predictive value = percent of patients designated “not at risk” by the tool who did not experience the outcome. Generalized linear models with log link for the regression of each observed clinical outcome on the risk assessment score are also presented. The table contains estimates and 95% confidence intervals. RR = relative risk (aka risk ratio), ref. = reference.

Abbreviations: 95% CI = 95% confidence interval, AWHONN = Association of Women’s Health, Obstetrics and Neonatal Nurses, GLM = Generalized Linear Model, NPV = negative predictive value, PPV = positive predictive value.

When using the WHO definition of PPH as blood loss ≥ 500 mL (second column section of the tables), the sensitivity is low for both the CMQCC and the ACOG tools. The sensitivity is highest for the AWHONN risk tool, which as noted scored many more participants as high or medium risk, particularly when both high and medium risk are considered, with 86.0% (95% CI: 83.0%–89.0%) of PPH cases correctly identified by the scale. The AWHONN’s high‐ and medium‐risk groups also had the highest negative predictive value (78.7%, CI: 75.2–82.2).

When using the ACOG definition of PPH as blood loss ≥ 1000 mL (third column section of the tables), the performances of the four predictors improve. The AWHONN risk assessment shows the strongest sensitivity (and worst specificity) of all the risk assessments, particularly when both high and medium risk levels are used to predict PPH. The sensitivity of the AWHONN indicates that 92.7% (95% CI: 90.5%–94.9%) of cases with ≥ 1000 mL blood loss were correctly predicted by the AWHONN. The negative predictive value (95.3%, CI: 93.5%–97.1%) indicates that only about 5% of cases that were classified by the AWHONN as “low risk” went on to experience a blood loss of ≥ 1000 mL (*n* = 6 cases). As a trade‐off, however, the AWHONN has poor specificity and poor positive predictive value and a high rate of false positives. The positive predictive value, for instance, shows that only 19.1% (CI 158%–22.5%) of the cases that AWHONN identified as “medium” or “high” risk experienced blood loss ≥ 1000 mL.

Table 3 also presents the results of generalized linear models with log links, examining each of the four risk assessments as predictors of observed clinical medication use and blood loss volumes. Hemoglobin values below 10 were not significantly associated with the administration of medication (RR 1.2, 95% CI 0.7–2.1), but were significantly associated with both blood loss volumes.

Figure 1 shows the ROC for each risk assessment tool, across the three observed clinical outcomes. For uterotonic administration, the areas under the curve for CMQCC (0.62, 95% CI 0.57–0.67) and AWHONN (also 0.62, 95% CI 0.57–0.67) are in the range that is commonly considered “poor discrimination,” and the areas for ACOG (0.58, 95% CI 0.53–0.63) and hemoglobin (0.51, 95% CI 0.48–0.53) are in the “failure” or “very poor” range. For blood loss, the areas under the curve remain poor for the three scales (ranging from 0.62 to 0.67) and in the failure range for hemoglobin level (0.53, 0.55).

FIGURE 1Receiver operating characteristic (ROC) curves, with area estimates and 95% confidence intervals, for each risk assessment’s ability to predict actual medication administration or blood loss, plotting true positive rates (sensitivity) against false positive rates (1‐specificity). (a) Predicting actual uterotonic medication administration. (b) Predicting ≥ 500 mL blood loss. (c) Predicting ≥ 1000 mL blood loss.

**Figure:**
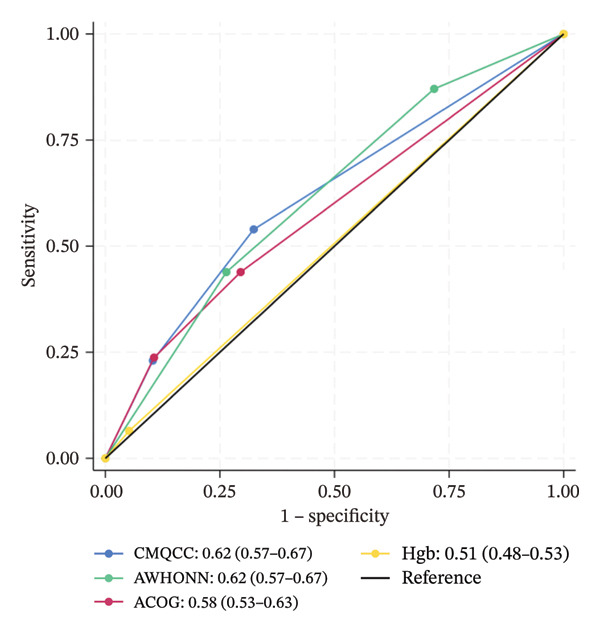
(a)

**Figure:**
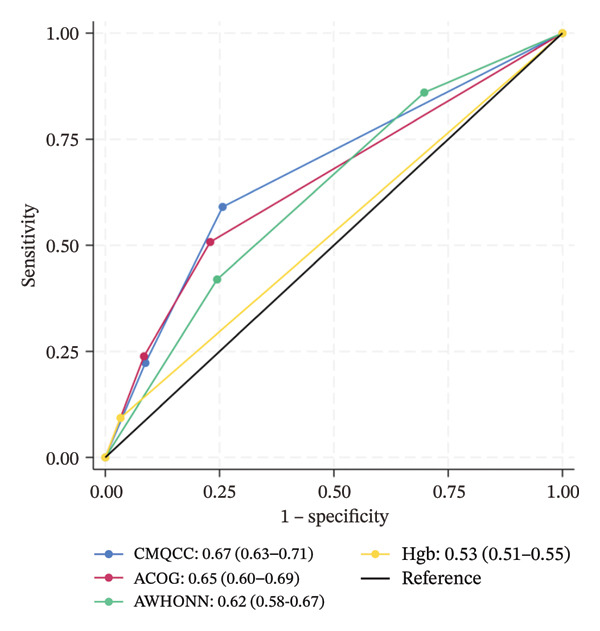
(b)

**Figure:**
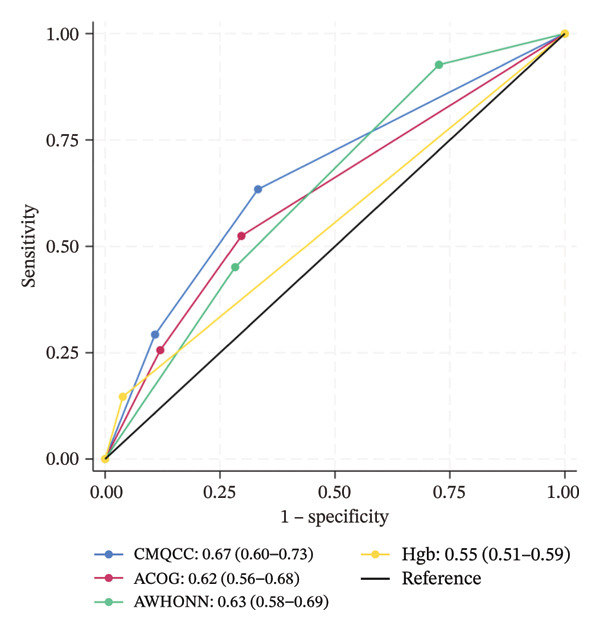
(c)

Figure 2 presents calibration plots, graphing the percent of participants in each risk group who experienced the clinical outcome. All tools trend in an appropriate direction, with more clinical outcomes occurring in higher‐risk groups. For ≥ 500 mL blood loss (Figure 2(b)), the CQMCC and ACOG tools do not separate well between medium and high risk, but separation is better for all tools at ≥ 1000 mL blood loss (Figure 2(c)). Since the AWHONN screening tool classified a larger proportion of participants as medium or high risk, the percent of participants scored medium or high risk by this tool who experienced blood loss or received medication appears consistently lower than other tools.

FIGURE 2Calibration plots showing the observed percentage of participants at each risk level who (a) received uterotonic medication, (b) experienced ≥ 500 mL blood loss, or (c) experienced ≥ 1000 mL blood loss. Reference lines appear at the overall percentage of participants experiencing the outcome for the whole sample (*n* = 525).

**Figure:**
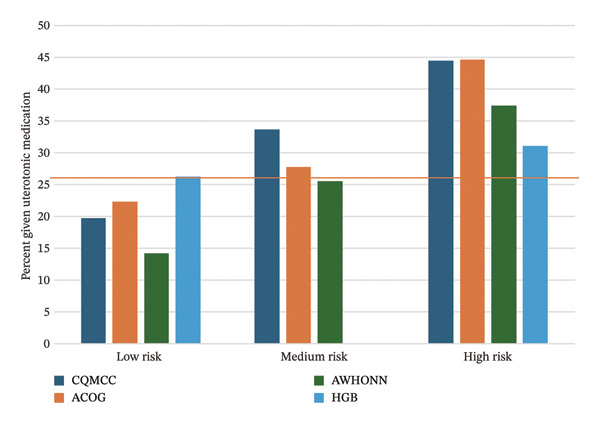
(a)

**Figure:**
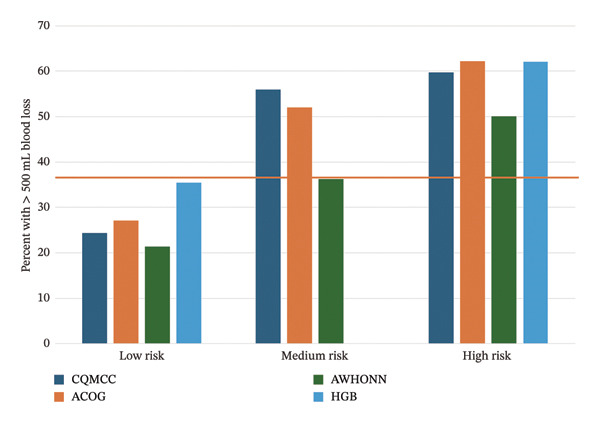
(b)

**Figure:**
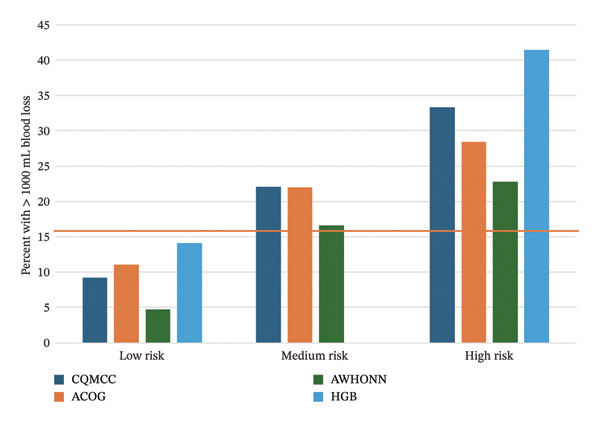
(c)

When participants who received uterotonic medications were excluded, sensitivity analyses showed similar patterns of sensitivity and specificity for blood loss outcomes (Supporting Table S2), similar ROC curves (Supporting Figure S1), and similar calibration (Supporting Figure S2).

## 4. Discussion

### 4.1. Main Findings

Our findings suggest that a significant proportion of hemorrhages are missed by the assessment tools, necessitating a more accurate solution to prepare birthing teams, curtail blood loss, and reduce severe patient morbidity and mortality. While the AWHONN tool’s high‐ and medium‐risk groups had the highest negative predictive values, it still failed to identify some cases that went on to experience high blood loss. Of the cases that the AWHONN tool identified to be low‐risk, 21% experienced blood loss of ≥ 500 mL (NPV 78.7%, CI 75.2%–82.2%) and 5% experienced a blood loss of ≥ 1000 mL (NPV 95.3%, CI 93.5%–97.1%). The AWHONN tool trades sensitivity for specificity, with many participants being identified as high or medium risk and a high rate of false positives. This may be clinically preferable to a high rate of false negatives. Since the AWHONN is not discriminating well between patients with blood loss and patients without, or between patients receiving uterotonic medications and those not, the ROC values show poor discrimination.

When using the ACOG definition of PPH as blood loss ≥ 1000 mL, the performances of the assessment tools improve. Once again, the AWHONN risk assessment shows the strongest sensitivity (and worst specificity) of all the risk assessments, particularly when both high‐ and medium‐risk groups are used to predict PPH.

### 4.2. Strengths and Limitations

A strength of this study is that it was conducted at a tertiary care hospital within an academic medical center with a racially, ethnically, and socioeconomically diverse population. Limitations of this study include that it was a secondary data analysis performed with data from just one institution in the United States whose inclusion criteria excluded parturients who are known to be high risk for hemorrhage by any criteria: those with preterm gestation, multiple gestations, and intrauterine fetal demise Additionally, an adaptation of the AWHONN tool was used for clinical care at this institution, thus indicating a source of potential incorporation bias. Another limitation is that EBL was used to document blood loss in 96% of cases, with QBL being used in only 4% of cases. Additionally, the data was collected during the height of the COVID‐19 pandemic and therefore, may represent a period of time where data are not representative of typical rates of PPH, blood loss and use of uterotonics.

### 4.3. Interpretation in Light of the Evidence

This study demonstrates that all three of the PPH risk assessment tools that were evaluated fall short in their predictive capacity, leaving a substantial number of PPHs missed and designating many patients as “at risk” who do not experience PPH. Even though the AWHONN tool had the highest sensitivity and negative predictive values, 21% of cases that the AWHONN identified to be “low risk” experienced blood loss of ≥ 500 mL.

PPH risk assessment aims to promote a higher quality of patient care. Under‐preparedness for patients labeled as low risk who ultimately sustain a hemorrhage and anticipatory investment of energy and resources in patients deemed high risk who ultimately do not sustain a hemorrhage may create undue stress and resource strain. Improved identification of true PPH risk supports the functioning of obstetrical teams and timely treatment for PPH, thus reducing the morbidity, mortality, and costs associated with each PPH.

## 5. Conclusion

New methods of risk assessment need to be imagined and developed to better capture patients who are being missed. This holds the promise of improvements to resource allocation, staff and patient preparedness, and ultimately, reductions in maternal morbidity and mortality. Using data from one urban, tertiary care institution in the United States limits generalizability to rural and community hospitals in the United States, as well as to other countries. Further research needs to be done to assess the applicability of these tools in LMIC countries, where it can truly be a matter of life or death if clinicians have the ability to more accurately predict who is at true risk for PPH and the need to give birth in a facility with ample medications, blood supply, and availability of surgical procedures, if necessary [5]. The results reported in the VIBRANT paper [14] suggest a 2–8‐h window is possible to identify risk for life‐threatening uterine atony, thus conferring the ability of more timely transfer to facilities with appropriate resources for treatment. The landscape of approaches to risk assessment is beginning to evolve [17, 18]. Some researchers have recommended and are actively exploring the incorporation of intrapartum or non‐obstetric risk factors into risk assessment, adding weighted values to risk factors for better discrimination and revising best practices for ongoing re‐evaluation of risk assessment. Developing unique risk assessment tools for each mode of delivery may also improve sensitivity and specificity. More accurate, accessible resources for PPH prediction require a reimagining of the existing framework. The results could have far‐reaching implications for maternal health worldwide.

## Author Contributions

S.M. did the initial conceptualization, data curation, supervision, and writing of the original draft and review and editing of the final paper. M.N. did some of the analysis and contributed to writing of original draft and review and editing. M.B. performed formal data analysis and writing–review and editing. Y.N.R. performed the initial literature search and contributed to the writing of the original draft and final review and editing. J.W. contributed to conceptualization, analysis, and writing–review and editing. H.S. contributed to conceptualization, analysis, and writing–review and editing. K.K.T. contributed to conceptualization, methodology, data supervision, writing of the original draft, and writing–review and editing.

## Funding

Funding was obtained from University of Pennsylvania School of Nursing Innovation Accelerator Award. This work received funding from the Villanova University Falvey Library Scholarship Open Access Reserve (SOAR) fund.

## Ethics Statement

Ethics approval was received from the Institutional Review Board of the University of Pennsylvania on May 25, 2021, Protocol # 848588.

## Conflicts of Interest

S.M. and J.W. are co‐founders of Vasowatch, Inc. H.S. is a medical advisor to Vasowatch, Inc., and K.K.T. has been a consultant to Vasowatch, Inc. The other authors declare no conflicts of interest.

## Supporting Information

Additional supporting information can be found online in the Supporting Information section.

## Supporting information


**Supporting Information** Table S1: Sensitivity, specificity, and predictive values of Hgb levels for blood loss and use of uterotonics. Table S2: Sensitivity, specificity, and predictive values for those who did not receive uterotonics. Figure S1: Receiver operating characteristic (ROC) curves for those who did not receive uterotonics. Figure S2: Calibration plots of outcomes for the sensitivity sample of those who did not receive uterotonics.

## Data Availability

The data that support the findings of this study are not publicly available due to privacy or ethical restrictions.
