# Supplementary material for: Direct Comparison of Three Postpartum Hemorrhage Risk Assessment Tools: A Retrospective Secondary Data Analysis
Source: Obstet Gynecol Int. 2026 May 21;2026:9576600. doi: 10.1155/ogi/9576600 (PMC13191787; doi:10.1155/ogi/9576600)

**Direct comparison of three postpartum hemorrhage risk assessment tools: a retrospective secondary data analysis**

**Supplemental Materials**

**Table S1.** Sensitivity, specificity, and predictive values of Hgb levels, for predicting uterotonic medication administration (n=525), and blood loss volume (n=524). Generalized linear models with log link for the regression of each observed clinical outcome on the risk assessment score are also presented. The table contains estimates and 95% confidence intervals.

| **Hgb** | **Uterotonic Medication** | | | **Blood Loss ≥500ml** | | | | | **Blood Loss ≥1000ml** | | | | | |
| --- | --- | --- | --- | --- | --- | --- | --- | --- | --- | --- | --- | --- | --- | --- |
| **High Risk** | *%* | *(95%* | *CI)* | *%* | *(95%* | | *CI)* | | *%* | *(95%* | | *CI)* | |  |
| Sensitivity | 6.5 | (4.4% - | 8.6%) | 9.3 | (6.8% - | | 11.8%) | | 14.6 | (11.6% - | | 17.7%) | |  |
| Specificity | 94.8 | (92.9% - | 96.7%) | 96.7 | (95.1% - | | 98.2%) | | 96.2 | (94.5% - | | 97.8%) | |  |
| PPV | 31.0 | (27.1% - | 35.0%) | 62.1 | (57.9% - | | 66.2%) | | 41.4 | (37.2% - | | 45.6%) | |  |
| NPV | 73.8 | (70.0% - | 77.5%) | 64.7 | (60.6% - | | 68.7%) | | 85.9 | (82.9% - | | 88.8%) | |  |
| ***GLM*** | *RR* | *(95%* | *CI)* | *RR* | *(95%* | | *CI)* | | *RR* | *(95%* | | *CI)* | |  |
| (ref: Low) |  |  |  |  |  | |  | |  |  | |  | |  |
| High Risk | 1.2 | (0.7 - | 2.1) | 1.8 | (1.3 - | | 2.4) | | 2.9 | (1.8 - | | 4.8) | |  |
|  |  |  |  |  |  | |  | |  |  | |  | |  |
| ABBREVIATIONS: | |  |  |  |  | |  | |  |  | |  | |  |
| Hgb = hemoglobin; 95%CI = 95% confidence interval; | | | | | |  | |  |  | |  | |  | |
| PPV= positive predictive value; NPV= negative predictive value; RR = relative risk (aka risk ratio) | | | | | | | | | | | | | | |
| GLM= Generalized Linear Model; ref.= reference | | | | | | | | | | | | | | |
| NOTE: |  |  |  |  |  | |  | |  |  | |  | |  |
| Sensitivity = percent of patients with the outcome who were designated “at risk” by the tool. | | | | | | | | | | | | | | |
| Specificity = percent of patients without the outcome who were designated “not at risk” by the tool. | | | | | | | | | | | | | | |
| Positive predictive value = percent of patients designated “at risk” by the tool who experienced the outcome. | | | | | | | | | | | | |  | |
| Negative predictive value = percent of patients designated “not at risk” by the tool who did not experience the outcome. | | | | | | | | | | | | | | |

**Table S2.** Sensitivity, specificity, and predictive values of CQMCC, ACOG, AWHONN, and hgb assessments, for the limited sample of participants who did not receive uterotonic medications (n=386). Generalized linear models with log link for the regression of each observed clinical outcome on each risk assessment score are also presented. The table contains estimates and 95% confidence intervals.

|  | **Blood Loss ≥500ml** | | | **Blood Loss ≥1000ml** | | |
| --- | --- | --- | --- | --- | --- | --- |
| **CQMCC "High risk"** | |  |  |  |  |  |
| Sensitivity | 18.63% | 14.74% | 22.52% | 22.22% | 18.07% | 26.38% |
| Specificity | 92.58% | 89.96% | 95.20% | 90.50% | 87.57% | 93.43% |
| PPV | 47.50% | 42.51% | 52.49% | 15.00% | 11.43% | 18.57% |
| NPV | 75.94% | 71.67% | 80.21% | 93.91% | 91.52% | 96.30% |
| **CQMCC "High or medium risk"** | | |  |  |  |  |
| Sensitivity | 58.82% | 53.91% | 63.74% | 62.96% | 58.14% | 67.79% |
| Specificity | 77.39% | 73.21% | 81.56% | 70.11% | 65.54% | 74.68% |
| PPV | 48.39% | 43.40% | 53.38% | 13.71% | 10.27% | 17.15% |
| NPV | 83.91% | 80.24% | 87.58% | 96.17% | 94.25% | 98.09% |
| ***GLM: CQMCC*** |  |  |  |  |  |  |
| (ref: Low) | *RR* | *(95%* | *CI)* | *RR* | *(95%* | *CI)* |
| Medium Risk | 3.03 | 2.13 | 4.32 | 3.42 | 1.50 | 7.76 |
| High Risk | 2.95 | 1.92 | 4.53 | 3.92 | 1.51 | 10.18 |
| **ACOG "High risk"** | |  |  |  |  |  |
| Sensitivity | 22.55% | 18.37% | 26.72% | 18.52% | 14.64% | 22.40% |
| Specificity | 93.64% | 91.20% | 96.08% | 89.94% | 86.94% | 92.95% |
| PPV | 56.10% | 51.14% | 61.05% | 12.20% | 8.93% | 15.46% |
| NPV | 77.03% | 72.83% | 81.24% | 93.60% | 91.16% | 96.05% |
| **ACOG "High or medium risk"** | | |  |  |  |  |
| Sensitivity | 53.92% | 48.94% | 58.90% | 55.56% | 50.59% | 60.52% |
| Specificity | 79.51% | 75.47% | 83.54% | 72.63% | 68.17% | 77.08% |
| PPV | 48.67% | 43.68% | 53.67% | 13.27% | 9.89% | 16.66% |
| NPV | 82.72% | 78.94% | 86.50% | 95.59% | 93.54% | 97.64% |
| ***GLM: ACOG*** |  |  |  |  |  |  |
| (ref: Low) | *RR* | *(95%* | *CI)* | *RR* | *(95%* | *CI)* |
| Medium Risk | 2.57 | 1.78 | 3.71 | 3.15 | 1.42 | 6.99 |
| High Risk | 3.25 | 2.23 | 4.73 | 2.76 | 1.03 | 7.44 |
| **AWHONN "High risk"** | |  |  |  |  |  |
| Sensitivity | 41.18% | 36.26% | 46.09% | 40.74% | 35.83% | 45.65% |
| Specificity | 79.15% | 75.09% | 83.21% | 74.86% | 70.53% | 79.19% |
| PPV | 41.58% | 36.66% | 46.51% | 10.89% | 7.78% | 14.00% |
| NPV | 78.87% | 74.80% | 82.95% | 94.37% | 92.06% | 96.67% |
| **AWHONN "High or medium risk"** | | |  |  |  |  |
| Sensitivity | 84.31% | 80.68% | 87.95% | 92.59% | 89.98% | 95.21% |
| Specificity | 32.86% | 28.17% | 37.55% | 29.89% | 25.32% | 34.46% |
| PPV | 31.16% | 26.53% | 35.79% | 9.06% | 6.19% | 11.92% |
| NPV | 85.32% | 81.79% | 88.86% | 98.17% | 96.82% | 99.51% |
| ***GLM:AWHONN*** | |  |  |  |  |  |
| (ref: Low) | *RR* | *(95%* | *CI)* | *RR* | *(95%* | *CI)* |
| Medium Risk | 1.70 | 1.17 | 2.48 | 4.36 | 1.01 | 18.81 |
| High Risk | 2.35 | 1.63 | 3.40 | 5.94 | 1.35 | 26.13 |
| **Hgb Risk** |  |  |  |  |  |  |
| Sensitivity | 10.78% | 7.69% | 13.88% | 18.52% | 14.64% | 22.40% |
| Specificity | 96.82% | 95.07% | 98.57% | 95.81% | 93.81% | 97.81% |
| PPV | 55.00% | 50.03% | 59.97% | 25.00% | 20.67% | 29.33% |
| NPV | 75.07% | 70.75% | 79.39% | 93.97% | 91.60% | 96.35% |
| ***GLM: HGB*** |  |  |  |  |  |  |
| (ref: Low) | *RR* | *(95%* | *CI)* | *RR* | *(95%* | *CI)* |
| High Risk | 2.21 | 1.43 | 3.41 | 4.15 | 1.75 | 9.81 |
|  |  |  |  |  |  |  |
| ABBREVIATIONS: | |  |  |  |  |  |
| RR = relative risk (aka risk ratio); 95%CI = 95% confidence interval; CMQCC = California Maternal Quality Care Collaborative; PPV= positive predictive | | | | | | |
| value; NPV= negative predictive value; ACOG = American College of Obstetricians and Gynecologists; | | | | | | |
| AWHONN = Association of Women’s Health, Obstetrics and Neonatal Nurses; Hgb = hemoglobin | | | | | | |
| NOTE: |  |  |  |  |  |  |
| Sensitivity = percent of patients with the outcome who were designated “at risk” by the tool. | | | | | | |
| Specificity = percent of patients without the outcome who were designated “not at risk” by the tool. | | | | | | |
| Positive predictive value = percent of patients designated “at risk” by the tool who experienced the outcome. | | | | | | |
| Negative predictive value = percent of patients designated “not at risk” by the tool who did not experience the outcome. | | | | | | |
|  |  |  |  |  |  |  |

**Figure S1.** Receiver operating characteristic (ROC) curves for sensitivity analysis including only patients who did not receive any uterotonic medications (n=386). Plots include area estimates and 95% confidence intervals for each risk assessment’s ability to predict blood loss, plotting true positive rates (sensitivity) against false positive rates (1-specificity).

1. Predicting ≥500mL blood loss

**
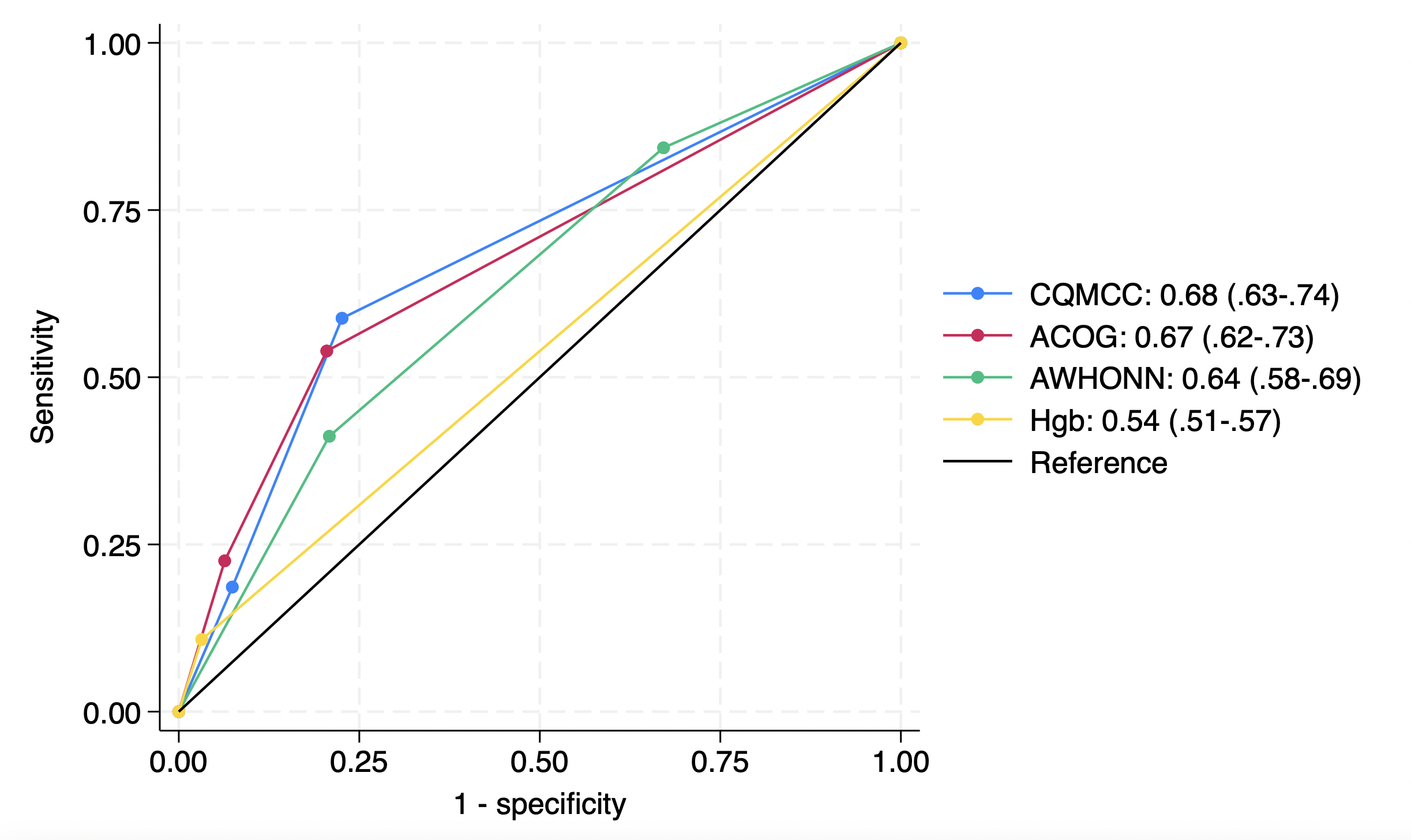
**

1. Predicting ≥1000mL blood loss


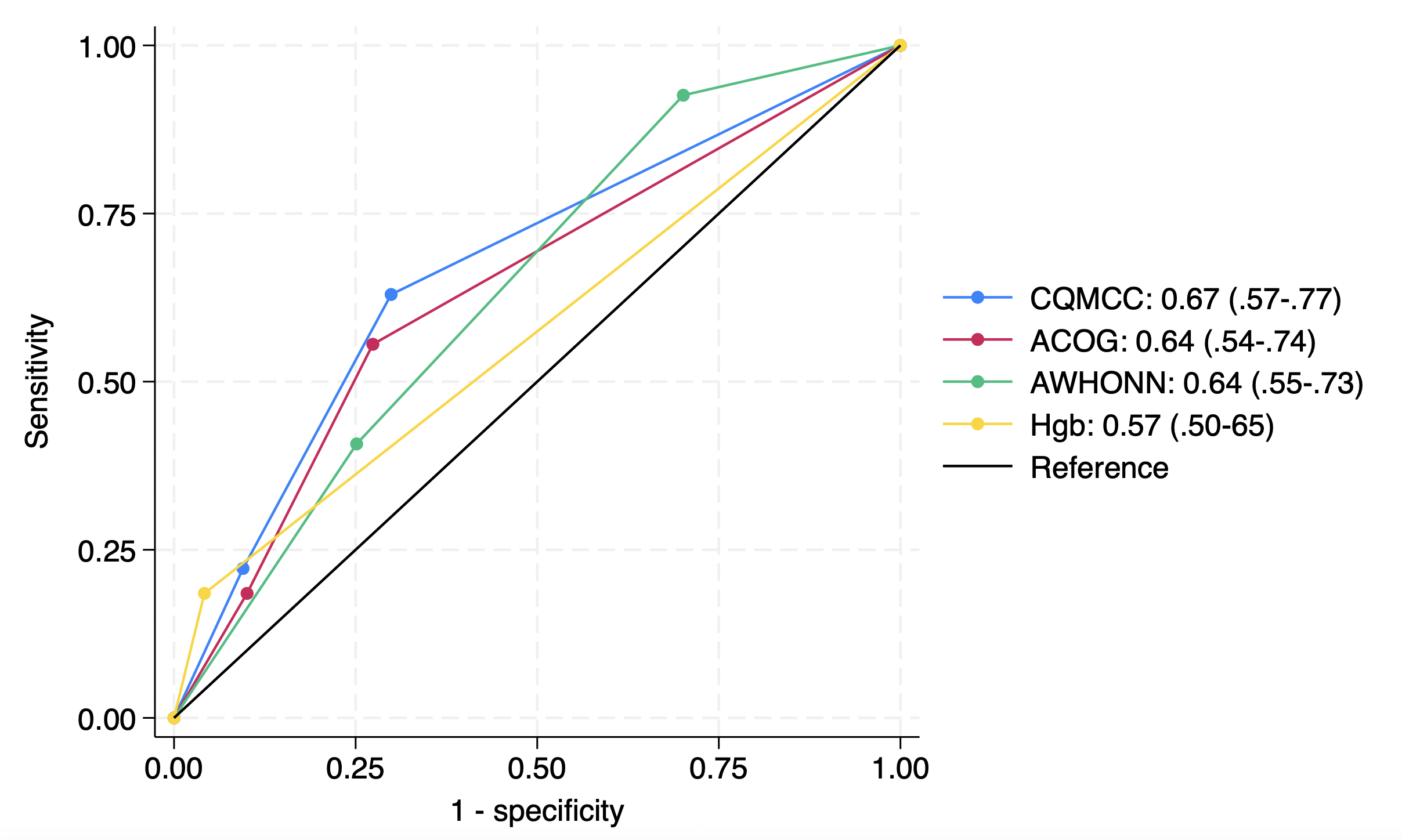


**Figure S2.** Calibration plots for the sensitivity sample (n=386) showing the observed percentage of participants at each risk level who (a) experienced ≥500ml blood loss, or (b) experienced ≥1000ml blood loss. Reference lines appear at the overall percentage of participants experiencing the outcome for this sample who did not receive uterotonic medication.

1. Percent in each risk group who experienced ≥500ml blood loss (26.5% overall)


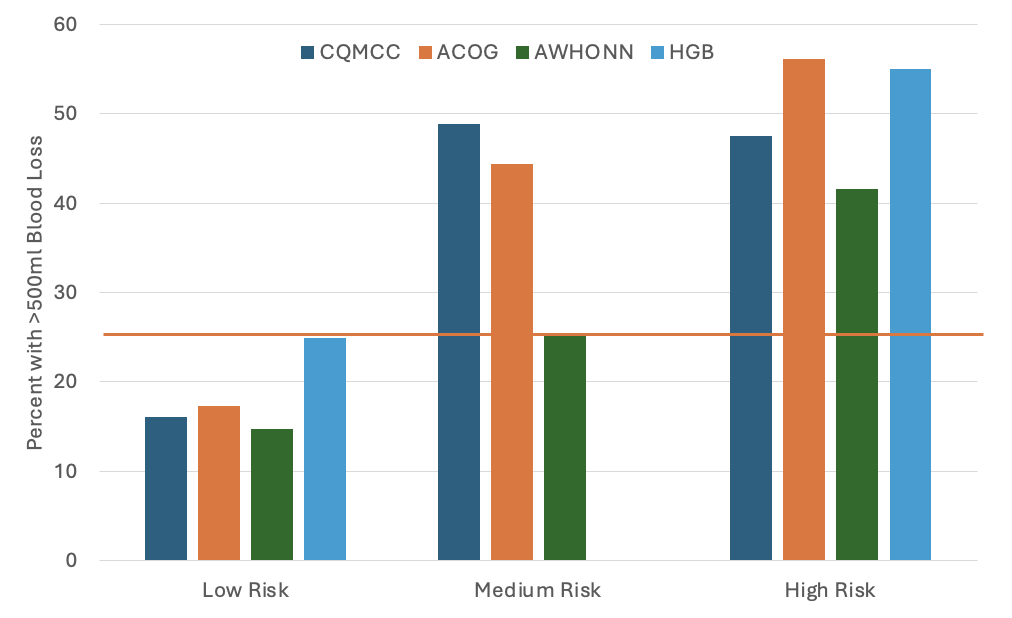


1. Percent in each risk group who experienced ≥1000ml blood loss (7.0% overall)


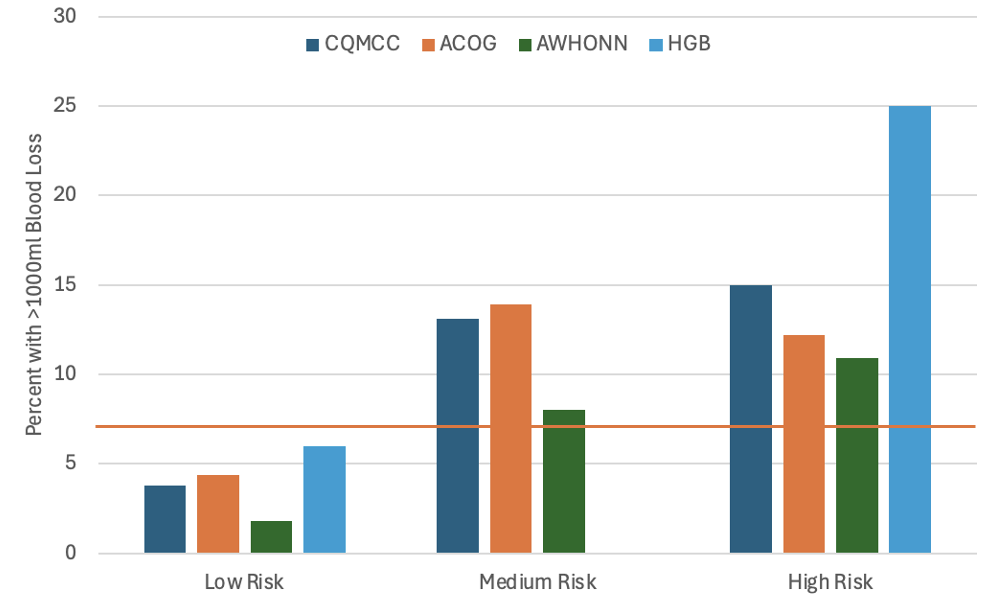

Supplement: Supplementary file 1 — Supporting Information Table S1: Sensitivity, specificity, and predictive values of Hgb levels for blood loss and use of uterotonics. Table S2: Sensitivity, specificity, and predictive values for those who did not receive uterotonics. Figure S1: Receiver operating characteristic (ROC) curves for those who did not receive uterotonics. Figure S2: Calibration plots of outcomes for the sensitivity sample of those who did not receive uterotonics. [file OGI-2026-9576600-s001.docx]
